# Bioaccessibility and antioxidant capacity of kefir‐based smoothies fortified with kale and spinach after in vitro gastrointestinal digestion

**DOI:** 10.1002/fsn3.3917

**Published:** 2024-01-08

**Authors:** Lutfiye Yilmaz‐Ersan, Tulay Ozcan, Buse Usta‐Gorgun, Melike Ciniviz, Gokce Keser, Ilay Bengu, Raziye Asli Keser

**Affiliations:** ^1^ Faculty of Agriculture, Department of Food Engineering Bursa Uludag University Bursa Turkey; ^2^ Graduate School of Natural and Applied Sciences Bursa Uludag University Bursa Turkey

**Keywords:** antioxidant, bioaccessibility, kale, kefir, smoothie, spinach

## Abstract

The kefir‐based smoothies with kale and spinach were designed as a ready‐to‐drink and innovative functional snack. Microbiological, physicochemical, as well as pre‐ and postgastrointestinal total antioxidant capacity (TAC; CUPRAC, DPPH, and FRAP) analyses were conducted. It was determined that the kefir‐based smoothies with vegetables had higher ash, carbohydrate, and dietary fiber values. Fructose and glucose contents of smoothy with kale were high, while smoothy with spinach included high sucrose and maltose. The microbiology results revealed that kefir‐based vegetable smoothies had minimum Lactobacillaceae viability (>log 7 cfu g^−1^) for the required functional effect after 14‐day storage. Moreover, the addition of kale significantly increased (*p* < .01) the level of initial TAC (CUPRAC, DPPH, and FRAP) and total phenolic content (TPC) values. After in vitro gastric digestion analysis, smoothie with spinach demonstrated higher TAC and TPC values and the control sample had higher TAC and TPC values compared with a predigestion step. It was found that in vitro intestinal DPPH values were higher for the sample with spinach samples, while the sample with kale had the highest FRAP values. It was also found that the bioaccessibility indexes of plain kefir were determined to be the highest in both in vitro gastric and intestinal procedures. The present study provided novel insights into the in vitro digestion properties of kefir fortified with vegetables. Nevertheless, further studies are needed to identify the functional properties of the milk and plant matrices mixture using in vitro and in vivo trials.

## Graphical abstract

The kefir‐based smoothies were designed as a ready‐to‐drink. Fortification with vegetables improved the nutritional quality of samples. The addition of kale increased initial antioxidative properties. Bioaccessibility indexes of plain kefir were determined to be the highest in vitro gastrointestinal digestion. CUPRAC, DPPH, and FRAP values changed depending on gastric and intestinal conditions.

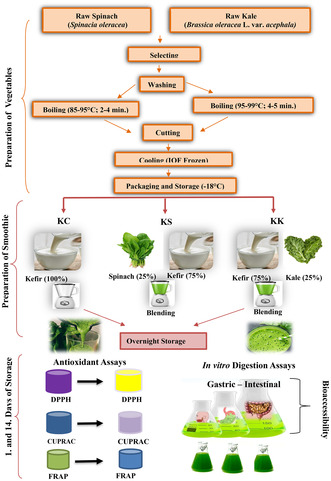

## 1 INTRODUCTION

Nowadays, consumers have a tendency to focus on ready‐to‐eat foods in an attempt to reduce the time required for preparing food due to globalization, industrialization, and the changing lifestyles instigated by modern society. In this sense, smoothies, ready to consume, have been increasingly becoming one of the emerging trends in order to meet consumers' specific needs and daily nutrition. Smoothies are semiliquid beverages with a smooth consistency. They are formulated using fruit, fruit juice/puree, vegetable, vegetable juice/puree, milk, yogurt, kefir, ice cream, sodas, tea, lemonade, condiments, spices, and honey (Ajlan et al., 2022; Cano‐Lamadrid et al., 2020; da Silva‐Mojón et al., 2023). Smoothies are excellent, convenient, and refreshment beverages that promote the daily consumption of either animal‐ or plant‐based foods. In addition to being sold commercially in markets, smoothies can also be prepared practically at home by consumers who demand healthy ingredients, clean labels, and minimally processed foods. Smoothies are accepted as key drivers for ready‐to‐beverage market growth due to the growing global trends. According to The International Market Analysis Research and Consulting Group (IMARC Group), the market size of smoothies was valued at US$ 15.7 Billion in 2022 and is estimated to achieve US$ 21.1 Billion by 2028 with a compound annual growth rate of 4.7% (Castillejo et al., 2016; IMARC Group, 2023; Krahulcová et al., 2021; McCartney et al., 2018, 2019).

In this respect, kefir can be preferably utilized as the best base for smoothies due to its high nutrient density (protein, Ca, Mg, K, Zn, and P) and wealthy bioactive compounds such as essential amino acids, peptides, lactic acid bacteria, conjugated linoleic acid, and organic acids. Kefir is a fermented dairy beverage with a slightly carbonated, refreshing, and acidic taste. A starter culture composed of lactic acid bacteria, acetic acid bacteria, yeasts, and mycelial fungi is used for kefir production. It is claimed that kefir consumption on a daily basis provides antimicrobial, anti‐inflammatory, antitumor, and antioxidative effects, and helps lactose intolerance and cholesterol metabolism (Azizi et al., 2021; Yilmaz et al., 2022).

Vegetables have a vital role to play due to their high nutritional content and health‐promoting values. Furthermore, the World Health Organization recommends the consumption of at least 400 g of nonstarchy vegetables and fruits for daily nutritional requirements. As far as vegetables are concerned, it was stated that vegetables, such as dark‐green leafy vegetables including kale, broccoli, and spinach, have been stated to have excellent content of micro–macronutrients as well as bioactive phytochemicals. Kale (*Brassica oleracea* var. *acephala*), Greek cauliflower, belongs to the cabbage family of Brassicaceae, while Spinach (*Spinacea oleracea*) is a member of the Chenopodiaceae family. There has recently been an increasing demand for both vegetables since kale contains high amounts of prebiotic compounds and spinach contains all the essential amino acids to be taken daily as recommended by the FAO nutritional standards. Both kale and spinach manifest positive health benefits including anticarcinogen, antidiabetic, antihypertensive, anti‐inflammatory, atheroprotective, gastroprotective, neuroprotective, reduction in cholesterol absorption, and prevention of bone mineral density (Agarwal et al., 2017; Maqbool et al., 2021; Natesh et al., 2017; Ortega‐Hernández et al., 2021; Papierska et al., 2022; Šamec et al., 2019; Satheesh & Fanta, 2020; Thakur et al., 2022).

Fermented dairy‐based smoothies with vegetables and fruits may cause an increase in the concentration of bioactive compounds providing a high nutritional value and positively contributing to therapeutic activities, wellbeing, and a healthy lifestyle (Gallina et al., 2019; Gallina & Barbosa, 2022; McCartney et al., 2019; Miano et al., 2021). Despite the numerous studies on the microbiological, physicochemical, rheological, antioxidative, and sensorial properties of kefir fortified with various plants (da Costa et al., 2018; Kabakci et al., 2020; Kim et al., 2022; Paredes et al., 2022; Vimercati et al., 2020; Yilmaz et al., 2006), in the relevant research literature, it was determined that kefir‐based smoothies were not studies as ready‐to‐beverage. Nevertheless, information regarding the bioavailable and bioaccessible of these products has insufficiently been focused on. Bioavailability is defined as the fraction of ingested nutrients that are released from the food matrix and reach the relevant sites of the body in an active form. Bioavailability is affected by the composition, pH, structure, rheology, and processing of foods as well as molecular weight, solubility, hydrophobicity, proton donor/acceptor, and physicochemical attributes of bioactives. The determination of bioavailability requires in vivo experiments that are expensive and require extended periods of time and ethical restrictions. Bioaccessibility, that is, the scope of bioavailability, is an essential first experiment for better identification of the biological properties of food components. The bioaccessibility of food components in many studies is assessed through in vitro methodology, which is performed in gastric with pepsin enzyme and intestinal with pancreatin enzyme model system. When compared with bioavailability, bioaccessibility assays are cheap, reproducible, and fast methods (Dima et al., 2020; Rodrigues et al., 2022). Taking the above into account, kefir‐based smoothies with kale and spinach were designed as a ready‐to‐beverage and innovative functional snack. The present study was undertaken in order to evaluate the microbiological, physicochemical, and antioxidative properties and to identify the bioaccessibility of the antioxidative compounds of samples during the in vitro gastrointestinal process.

## 2 MATERIALS AND METHODS

### 2.1 Materials

Kefir was purchased from the Eker Dairy Products Food Industry and Trade Inc. (Bursa, Turkiye). Individually quick‐frozen (IQF) diced kale (*B. oleracea* L. var. *acephala*; 10 × 10 mm) and IQF‐diced spinach (*Spinacia oleracea*; 10 × 10 mm) harvested from the Manisa province (Turkiye) were obtained from Martas Marmara Agricultural Food Products Inc. (Bursa, Turkiye). The gross composition based on physico‐chemical properties of kefir and vegetables by determined producing companies is illustrated in Table 1.

**TABLE 1:** The gross composition of kefir and vegetables used in production.

Composition	Kefir (g 100 mL^−1^)	IQF diced kale (g 100 g^−1^)	IQF diced spinach (g 100 g^−1^)
Dry matter	9.57	9.39	7.79
Protein	2.70	2.98	2.95
Fat	3.20	0.52	0.39
Ash	0.50	1.18	1.07
Carbohydrate	3.20	2.24	1.32
Dietary fiber	0.47	2.47	2.06

Abbreviation: IQF, individually quick frozen.

### 2.2 Preparation of kefir‐based smoothies fortified with kale and spinach

**FIGURE 1:**
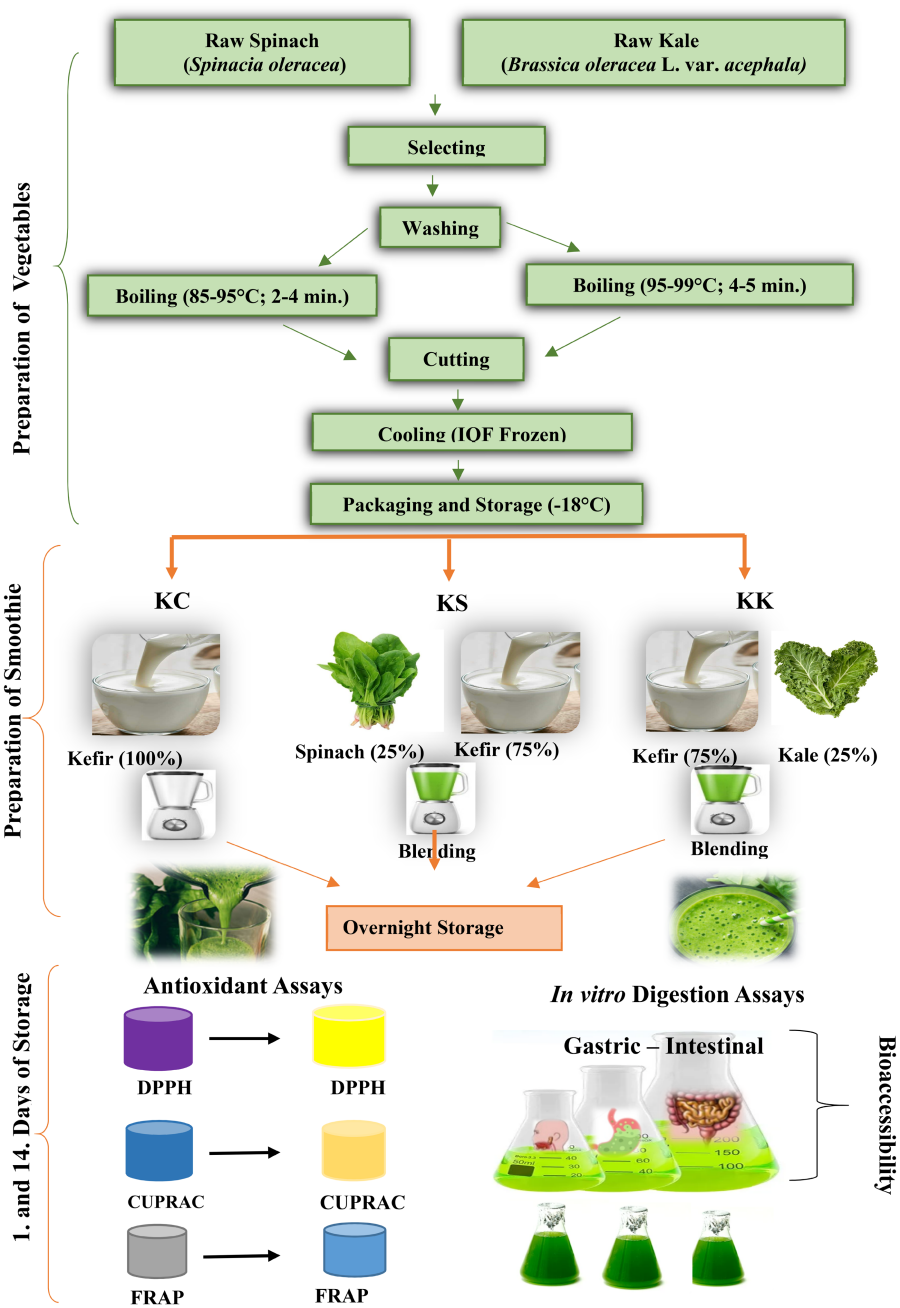
Preparation of kefir‐based smoothies fortified with kale and spinach. KC, plain kefir; KK, kefir‐based smoothie with kale; KS, kefir‐based smoothie with spinach.

The preparation scheme for kefir‐based smoothies fortified with kale and spinach is illustrated in Figure 1. Kefir was fortified with frozen kale and spinach at 1:4 w/v concentration. Kefir and vegetables were aseptically blended in a homogenizer for 30 s. The plain kefir, kefir‐based smoothie with kale, and kefir‐based smoothie with spinach were designated KC, KK, and KS, respectively. Kefir‐based smoothies were filled into sterilized glass bottles and stored at 4 ± 1°C. Samples were analyzed in a 1‐week time period during storage.

### 2.3 Microbiological assays

The enumeration of total mesophilic aerobic bacteria (TMAB) was assessed using the plate count agar (PCA, Merck, USA) and incubated at 30°C for 72 h. To determine Lactobacillaceae counts, the MRS (De Man, Rogosa, and Sharpe agar, Merck, Germany) medium and M17 medium (Merck, Germany) were used. MRS agar plates were incubated at 30°C under anaerobic conditions (Anaerocult C, Merck, Germany) for 72 h and M17 agar plates were incubated in the same conditions for 48 h (Irigoyen et al., 2005; Zhi et al., 2021). The yeast glucose chloramphenicol agar (YGCA, Merck, Germany) and *Acetobacter peroxydans* medium were used for yeast counts and acetic acid bacteria (AAB) counts, respectively. Both of them were incubated aerobically for 3–5 days at 25°C based on the way it was described by Witthuhn et al. (2005). Enterobacteriaceae counts were established using violet red bile agar (VRBA, Merck, Germany) and incubated at 37°C for 24 h (Fernandez et al., 2018). All results were enumerated as a log of colony‐forming unit (CFU) gram of the sample.

### 2.4 Physicochemical assays

The pH of the samples (Hanna pH 211 pH meter, Hanna Instruments, USA) was measured. The syneresis was implemented as the whey (mL) filtered (Whatman No 1 Filter Paper, Merck, Darmstadt, Germany) per 25 g sample at 4°C for 2 h. The proximate components of samples were analyzed: dry matter, protein, fat, and ash according to the AOAC methods (AOAC, 2016) and dietary fiber by the enzymatic–gravimetric method (AOAC, 2005). The proximate carbohydrate amount was calculated by subtracting the percentage of moisture, total protein, total fat, and total ash. To analyze sugars, 5‐g samples were suspended in 10 mL of mixed methanol/water (25:75, v/v), and then filtered using a 0.45 μm membrane filter (Sigma‐Aldrich, Taufkirchen, Germany). Sugars (fructose, glucose, saccharose, and maltose) were determined on high‐performance liquid chromatography (HPLC, 20ACBM, Shimadzu Prominence, Japan) equipped with a refractive index detector (RID‐10 A). The HPLC consisted of a Shimadzu CTO‐10ASvp column, a Shimadzu LC‐20AT pump, and a Shimadzu SIL‐20A/20AC autosampler. The separation was carried out on an Inertsil NH2 column (5 μm × 250 mm × 4.6 mm, Japan). The mobile phase was used as a mix of methanol 80% and water 20% with 1 mL min^−1^ flow rate. The column temperature was set at 30°C. A flow rate was adjusted to 0.8 mL min^−1^. Peak retention times describing the pure standard of each sugar were defined. Retention time (RT), the limit of detection (LOD), and the coefficient of correlation (*R* ^2^) for fructose were 7.7, 5.5, and .9999, respectively. RT, LOD, and *R* ^2^ for glucose were 8.1, 3.9, and .9996, respectively. RT, LOD, and *R* ^2^ for saccharose were 8.9, 4.8, and .9993, respectively. RT, LOD, and *R* ^2^ for maltose were 11.2, 3.9, and .9996, respectively. Sugar values calculated as ppm (mg L^−1^) were converted to g 100 mL^−1^ (Sousa et al., 2015). Calibration curves and chromatogram for fructose, glucose, saccharose and maltose of samples were supplied as supplementary file (Figures S1–S5).

### 2.5 Total antioxidant capacity (TAC) and total phenolic content (TPC) assays

The antioxidant capacity of the samples was identified with three complementary assays to provide reliable results: copper (II)‐ion reducing antioxidant capacity (CUPRAC; Apak et al., 2004), 2,2‐diphenyl‐1‐picryhydrazyl radical scavenging activity (DPPH; Kumaran & Karunakaran, 2006), and ferric reducing antioxidant power (FRAP; Benzie & Strain, 1996) assay. The results of TAC assays were expressed as mg Trolox equivalent (TE) per 100 mL of the sample. Total phenolic content was estimated by using Folin–Ciocalteu method according to the procedure reported by Sahin et al. (2013). TPC was represented in mg of gallic acid equivalents (GAE) per 100 mL of the sample. TAC and TPC assays were carried out in triplicate. Calibration curves and equations with R^2^ for TAC and TPC assays of samples were supplied as supplementary file (Figures S6–S9).

### 2.6 In vitro digestion assay

The in vitro gastrointestinal digestion assays were performed based on the way it was described by Minekus et al. (2014). The digestion conditions of gastric and intestinal medium were simulated (Table 2). The simulated gastric medium was composed of gastric fluids, pepsin (25,000 U mL^−1^, Sigma‐Aldrich P7012, USA), and HCl. The simulated gastric medium included intestinal fluids, pancreatin (800 U mL^−1^, Sigma‐Aldrich P7545, USA), bile (160 mM), and NaOH. For both mediums, incubation was performed at 37°C for 2 h in a shaking water bath. The mixing of sample and intestinal fluids was joined with 100 μL formic acid; afterward, the mixes were centrifuged at 10,000*g* for 10 min at 4° using the Sigma 3K30 centrifuge (Germany). The supernatants were filtered via 0.45 μm (MF‐Millipore® Membrane Filter, Sigma‐Aldrich, USA) and assayed for TAC and TPC.

**TABLE 2:** Composition of simulated gastric and intestinal fluids.

Chemicals (mmol L^−1^)	Simulated gastric fluids	Simulated intestinal fluids
	pH: 3.0	pH: 7.0
KCl (0.5 M)	6.9 mL	6.8 mL
KH_2_PO_4_ (0.5 M)	0.9 mL	0.8 mL
NaHCO_3_ (1 M)	12.5 mL	42.5 mL
NaCl (2 M)	11.8 mL	9.6 mL
MgCl_2_(H_2_0)_6_ (0.15 M)	0.4 mL	1.1 mL
(NH_4_)_2_CO_3_ (0.5 M)	0.5 mL	‐
CaCl_2_(H_2_O)_2_ (0.3 M)	1.3 mL	0.7 mL

The bioaccessibility index (BI%) for TAC and TPC was calculated as in the following equation.

$$\mathrm{BI}\;\text{for gastric}\;\left(\%\right)=\left(\frac{\mathrm{TAC}\;\text{and}\;\mathrm{TPC}\;\text{after the in vitro gastric digestion}}{\mathrm{TAC}\;\text{and}\;\mathrm{TPC}\;\text{before the in vitro gastric digestion}}\right)\times 100$$

$$\mathrm{BI}\;\text{for intestinal}\;\left(\%\right)=\left(\frac{\mathrm{TAC}\;\text{and}\;\mathrm{TPC}\;\text{after the in vitro intestinal digestion}}{\;\mathrm{TAC}\;\text{and}\;\mathrm{TPC}\;\text{before the in vitro intestinal digestion}}\right)\times 100$$

### 2.7 Statistical analysis

Statistical analyses were carried out using Minitab 17.0 for Windows (Minitab Inc., State College, PA). The results presented as means ± standard deviation were subjected to variance analysis (ANOVA) at 1% and 5% significance levels. The correlation matrix of the parameters analyzed was conducted using principal component analysis (PCA), which is the multivariate analysis method. Varimax with Kaiser normalization rotation method was used. Regression was chosen as a factor score (IBM SPSS Statistics 22.0 USA).

## 3 RESULTS AND DISCUSSION

### 3.1 Proximate composition and some physicochemical properties of the samples

According to FoodData Central Search Results, raw kale is composed of 4.4 g 100 g^−1^ of carbohydrates, 4.1 g 100 g^−1^ of fiber, 2.9 g 100 g^−1^ of protein, and 1.49 g 100 g^−1^ of lipids, while raw spinach includes 3.6 g 100 g^−1^ of carbohydrates, 2.2 g 100 g^−1^ of fiber, 2.9 g 100 g^−1^ of protein, and 0.4 g 100 g^−1^ of lipids (U.S. Department of Agriculture, Agricultural Research Service, 2022). The proximate composition is presented in Table 3. It was found that the differences among the gross composition of the samples were statistically significant (*p* ≤ .01). The dry matter values of the samples varied between 8.92 for KK to 9.57% for KC. Protein values of the samples including control, spinach, and kale were identified as 2.70%, 3.16%, and 2.90%, respectively. The fat contents varied between 0.95 for the smoothie with spinach and 3.20% for the control. The ash contents showed no significant difference (*p* > .05). The carbohydrate values ranged from 3.16% for control to 4.72 for the smoothie with spinach. The smoothie with spinach had higher saccharose and maltose contents, while higher fructose and glucose values were found in the sample with kale. Similarly, glucose, fructose, and sucrose of kale and spinach were identified as the major soluble sugars in the relevant studies (Proietti et al., 2023; Thavarajah et al., 2016). The dietary fiber contents of the samples were determined as being between 0.47% and 0.88%; the smoothies with spinach and kale had higher levels of dietary fiber. Generally, it was observed that the kefir‐based smoothies with vegetables had higher ash, protein, carbohydrate, glucose, and dietary fiber content. The differences in the composition of samples may be attributed to maturity, agro‐climatic conditions, agricultural practices, and frozen process of the vegetable species (Agarwal et al., 2017; Biondi et al., 2021; Satheesh & Fanta, 2020). Regarding vegetable‐based smoothies, the green vegetable smoothie including 6% spinach, 12% broccoli, and 77.2% cucumber was the subject of research by Castillejo et al. (2016), who reported that pH, titratable acidity, and dietary fiber content of the smoothie were 4.49, 0.22 mg citric acid 100 g^−1^, and 4.4% wet basis, respectively. The pH values (4.35–4.60) obtained in the present study were in line with the results reported by Castillejo et al. (2016), while dietary fiber values were found lower than researchers. As shown in Table 3, the type of sample, storage time, and their interaction significantly influenced the pH (*p* ≤ .01). During storage, the pH values of the control sample (KC) decreased, whereas those of the smoothie with kale increased. We observed unstable changes in the pH values of the sample with spinach. It was thought that the type and amount of organic acids formed depending on kefir microbiota activity during storage resulted in different pH changes in the smoothies. Paredes et al. (2022) determined that pH values of kefir fortificated with fruit and vegetable juice (70% apple, 9% strawberry, 12% carrot, and beet 9%) and fermented with different grain amounts (1%, 2%, 3%, and 4%) ranged from 3.39 ± 0.024 to 3.80 ± 0.041 during 48‐h fermentation. During the 7‐day storage, syneresis values of the samples did not differ significantly (*p* ≥ .05), and the smoothie with spinach showed higher values of syneresis than the other samples.

**TABLE 3:** Proximate composition and some physicochemical properties of the samples.

Proximate composition	Samples			Significance
	KC	KK	KS	
Dry matter (g 100 mL^−1^)	9.57 ± 0.002^a^	8.92 ± 0.203^b^	9.32 ± 0.106^ab^	*
Protein (g 100 mL^−1^)	2.70 ± 0.000^c^	2.90 ± 0.066^b^	3.16 ± 0.036^a^	**
Fat (g 100 mL^−1^)	3.20 ± 0.283^a^	1.43 ± 0.042^b^	0.95 ± 0.014^b^	**
Ash (g 100 mL^−1^)	0.50 ± 0026^a^	0.64 ± 0038^a^	0.71 ± 0.061^a^	NS
Carbohydrate (g 100 mL^−1^)	3.16 ± 0.304^b^	3.91 ± 0.283^a^	4.72 ± 0.148^a^	*
Dietary fiber (g 100 mL^−1^)	0.47 ± 0.020^b^	0.88 ± 0.040^a^	0.87 ± 0.070^a^	**
Fructose (mg 100 mL^−1^)	1.20 ± 0.000^b^	1.66 ± 0.006^a^	1.06 ± 0.003^c^	**
Glucose (mg 100 mL^−1^)	1.26 ± 0.011^c^	2.88 ± 0.045^a^	2.30 ± 0.022^b^	**
Sucrose (mg 100 mL^−1^)	2.22 ± 0.000^b^	2.22 ± 0.004^b^	2.34 ± 0.002^a^	**
Maltose (mg 100 mL^−1^)	0.09 ± 0.001^b^	0.09 ± 0.003^b^	1.32 ± 0.002^a^	**

Some physicochemical properties					*S*	*D*	*S* × *D*
	Days of Storage	KC	KK	KS			
pH	1	4.40 ± 0.010^cB^	4.53 ± 0.010^bB^	4.69 ± 0.010^aA^	**	**	**
	7	4.46 ± 0.010^bA^	4.53 ± 0.006^aB^	4.35 ± 0.006^cC^	**	**	**
	14	4.43 ± 0.006^bB^	4.60 ± 0.020^aA^	4.45 ± 0.000^bB^	**	**	**
Syneresis	1	10.67 ± 0.038^aB^	10.82 ± 0.015^aA^	10.76 ± 0.125^aA^	NS	*	NS
	7	10.91 ± 0.049^aA^	10.75 ± 0.074^aA^	9.90 ± 1.107^aA^	NS	NS	NS
	14	10.88 ± 0.012^bAB^	10.89 ± 0.006^bA^	10.95 ± 0.015^aA^	*	NS	NS

*Note*: Means with different lower letters are significantly different within a row (*p < .05; **p ≤ .01; NS, nonsignificant). Means with different lowercase and uppercase letters are significantly different within a row (among the samples) and within a column (among storage days) (*p < .05; **p ≤ .01; NS, nonsignificant) *S* = Samples; *D* = Storage days; *S* × *D* = Interaction between the samples and storage days.

Abbreviations: KC, plain kefir; KK, kefir‐based smoothie with kale; KS, kefir‐based smoothie with spinach.

### 3.2 Microbiological properties of the samples

Microbiological of the samples are presented in Table 4. During storage, the smoothies with spinach and kale had higher TMAB counts than the control. TMAB counts of these samples increased during the 7‐day storage. Lactobacillaceae on MRS agar and acetic acid bacteria counts were similar for all the samples, and statistical analysis showed that the sample type and storage time had an insignificant (*p ≥* .05) effect on these bacteria. Lactobacillaceae counts on M17 agar were similar (>8 cfu g^−1^) for the three types of smoothies on the first day of storage. The statistical analysis showed that storage time had a significant (*p* < .05) effect on the Lactobacillaceae counts on M17 agar of the KS sample. Kabakci et al. (2020) found that counts of Lactobacillaceae, *Lactococcus* spp., and yeast in kefir fortified with anthocyanin‐rich juices were between 3.96 and 7.61, 7.44 and 8.16, and 4.82 and 4.55 cfu g^−1^, respectively. Vimercati et al. (2020) revealed that the counts of Lactobacillaceae, mesophilic cocci, acetic acid bacteria, and yeast of coffee‐flavored kefir varied from 7.30 to 8.13, from 8.66 to 9.24, from 8.19 to 9.16, and from 6.34 to 6.78 cfu mL^−1^, respectively. Kefir must include a minimum of 7 cfu g^−1^ kefir microorganisms and 4 cfu g^−1^ yeast according to the Codex Standard 243 (2003). It was found that microbiology counts established in the present study were similar to the values recommended by the Codex Standard. Furthermore, the microbiology results reflected that kefir‐based vegetable smoothies had minimum cell viability for the desired therapeutic benefits. The sample type demonstrated an impact on the counts of Enterobacteriaceae (*p* ≤ .05), which increased (*p* ≤ .01) in the control and the smoothie with kale in comparison with the smoothie with spinach. There were significant differences (*p* ≤ .05) among the Enterobacteriaceae counts of the samples throughout storage. The higher Enterobacteriaceae counts were enumerated in the smoothie with spinach at all storage times. In a study by Castillejo et al. (2016), it was also pointed out that the Enterobacteriaceae counts of green vegetable‐based smoothies (77.2% cucumber, 12% broccoli, and 6% spinach) were incremented by 2.2–2.3 after 7‐day storage at 15°C. No yeast was detected in any of the samples in the present study (data not shown).

**TABLE 4:** Some microbiological properties of the samples.

Microorganism counts (log cfu g^−1^)	Days of storage	Samples			Significance		
		KC	KK	KS	*S*	*D*	*S* × *D*
TMAB	1	6.98 ± 0.000^aA^	6.80 ± 0.137^bB^	6.68 ± 0.285^cB^	NS	*	**
	7	6.44 ± 0.197^bB^	7.53 ± 0.204^aA^	8.05 ± 0.543^aA^	*	*	**
	14	6.52 ± 0.121^bB^	7.31 ± 0.048^aA^	7.37 ± 0.152^aA^	**	*	**
Lactobacillaceae on MRS agar	1	8.12 ± 0.163^aA^	8.00 ± 0.000^aA^	8.32 ± 0.228^aA^	NS	NS	NS
	7	7.58 ± 0.167^aA^	7.73 ± 0.133^aA^	8.23 ± 0.434^aA^	NS	NS	NS
	14	7.16 ± 0.447^aA^	7.58 ± 0.167^aA^	7.52 ± 0. 256^aA^	NS	NS	NS
Lactobacillaceae on M17 agar	1	8.76 ± 0.470^aA^	8.93 ± 0.253^aA^	8.48 ± 0.002^aB^	NS	NS	**
	7	7.74 ± 0.061^cB^	8.95 ± 0.000^bA^	9.80 ± 0.000^aA^	**	**	**
	14	7.90 ± 0.084^bA^	8.16 ± 0.061^abA^	8.52 ± 0.095^aA^	**	NS	**
AAB	1	8.07 ± 0.240^aA^	8.15 ± 0.000^aA^	7.90 ± 0.008^aA^	NS	NS	NS
	7	7.50 ± 0.707^aA^	7.98 ± 0.236^aA^	7.72 ± 0.000^aA^	NS	NS	**
	14	6.81 ± 0.159^bA^	7.41 ± 0.073^aA^	7.69 ± 0.083^aA^	**	NS	NS
Enterobacteriaceae	1	0.00 ± 0.000^cC^	3.66 ± 0.182^bAB^	4.23 ± 0.495^aA^	**	**	**
	7	3.09 ± 0.446^cB^	3.51 ± 0.028^bB^	5.03 ± 0.000^aA^	**	NS	**
	14	4.05 ± 0.000^aA^	3.94 ± 0.000^aA^	4.70 ± 0.000^aA^	**	*	**

*Note*: Means with different lowercase and uppercase letters are significantly different within a row (among the samples) and within a column (among storage days) (*p < .05; **p ≤ .01; NS, nonsignificant) *S* = Samples; *D* = Storage days; *S* × *D* = Interaction between the samples and storage days.

Abbreviations: AAB, acetic acid bacteria; KC, plain kefir; KK, kefir‐based smoothie with kale; KS, kefir‐based smoothie with spinach; TMAB, total mesophilic aerobic bacteria.

### 3.3 Total antioxidant capacity and total phenolic content of the samples

The main antioxidants in kale and spinach are characterized as the phenolic acids (e.g., gallic, chlorogenic, caffeic, hydroxycinnamic, sinapinic, and ferulic acids), carotenoids (e.g., lutein, zeaxanthin, and β‐carotene), flavonoids (e.g., kaempferol, quercetin, catechin, and epicatechin), hydrolysis products of glucosinolates (e.g., sinigrin, sulforaphane, thiocyanates, and isothiocyanates), and vitamins (e.g., C, E, and folate) (Ortega‐Hernández et al., 2021; Papierska et al., 2022; Šamec et al., 2019; Satheesh & Fanta, 2020). The type of sample, storage time, and their interaction significantly influenced the initial and simulated digestion TAC (CUPRAC, DPPH, and FRAP) and the TPC values (*p* < .01; Table 5). Undoubtedly, the fortification with green vegetables was denoted by the increased TAC and TPC smoothies, since significant differences were observed regarding kefir. In particular, the fortification of kale significantly increased (*p* < .01) the level of initial TAC (CUPRAC, DPPH, and FRAP) and the TPC values. Similarly, in some previous studies such as by Zhou and Yu (2006) and Sikora et al. (2008), it was also highlighted that kale had the highest antioxidant activity among some vegetables such as broccoli, spinach, and white and green cauliflower. Likewise, the control showed lower levels (*p* ≤ .01) of TAC and TPC values at all storage times. Regarding the storage days, the initial CUPRAC and FRAP values of all samples decreased, whereas the DPPH values of the smoothie with spinach and kale increased (*p* ≤ .01). As far as these results are concerned, it was found that the CUPRAC values of all samples were found higher than the DPPH and FRAP values. It is also explained by the fact that the FRAP method analyzed only the hydrophilic antioxidants (i.e., phenolic compounds, vitamin C, and sugars), the DPPH method analyzed only the lipophilic antioxidants (i.e., carotenoids, fatty acids, and tocopherols), whereas the CUPRAC method measured both the hydrophilic and lipophilic antioxidants. Furthermore, the differences among the TAC assays (CUPRAC, DPPH, and FRAP) could be attributed to the different reaction mechanisms and/or conditions (e.g., DPPH radical scavenging, copper (II) ion, and ferric reducing), quantification method, endpoint, and oxidant. The effect of storage time on the total phenolic amounts of the KC and KK samples was insignificant (*p* ≥ .05), whereas the values of KS samples demonstrated a decrease (*p* ≤ .01). In agreement with our study, Castillejo et al. (2016) revealed that the phenolic amounts of vegetable smoothies including cucumber, spinach, and broccoli decreased during storage. ABTS, FRAP, and DPPH values of the samples were determined by the same researchers as 395.7, 234.2, and 54.4 16.6 mg TE kg^−1^, respectively.

**TABLE 5:** Initial and in vitro digestion TAC and TPC values of the samples.

TAC	Days of storage	Samples			Significance		
		KC	KK	KS	*S*	*D*	*S* × *D*
Initial							
CUPRAC	1	24.61 ± 0.750^cA^	51.15 ± 1.631^aA^	36.86 ± 1.114^bA^	**	**	**
	14	21.74 ± 0.329^cB^	42.57 ± 0.835^aB^	24.70 ± 0.437^bB^	**	**	**
DPPH	1	2.51 ± 0.031^cA^	8.59 ± 0.042^aB^	6.74 ± 0.049^bB^	**	**	**
	14	2.46 ± 0.212^bA^	10.07 ± 0.108^aA^	9.69 ± 0.155^aA^	**	**	**
FRAP	1	0.57 ± 0.075^cA^	11.88 ± 0.223^aA^	4.76 ± 0.463^bA^	**	**	**
	14	0.19 ± 0.010^cB^	8.01 ± 0.574^aB^	3.25 ± 0.268^bB^	**	**	**
TPC	1	8.53 ± 0.811^cA^	20.12 ± 0.950^aA^	15.98 ± 0.185^bA^	**	**	**
	14	8.12 ± 0.157^cA^	17.42 ± 0.471^aA^	12.33 ± 0.543^bB^	**	**	**
Gastric digestion							
CUPRAC	1	27.83 ± 1.807^aB^	15.92 ± 1.732^bA^	28.08 ± 0.506^aB^	**	**	**
	14	34.42 ± 0.781^aA^	17.08 ± 1.136^cA^	31.67 ± 0.125^bA^	**	**	**
DPPH	1	7.95 ± 0.784^aB^	8.64 ± 0.657^aA^	8.80 ± 1.049^aB^	**	**	**
	14	13.30 ± 0.445^aA^	9.40 ± 0.370^bA^	13.21 ± 0.294^aA^	**	**	**
FRAP	1	2.49 ± 0.184^bA^	1.55 ± 0.096^cB^	3.28 ± 0.062^aA^	**	**	**
	14	3.27 ± 0.323^aA^	3.37 ± 0.352^aA^	2.69 ± 0.294^aA^	**	**	**
TPC	1	5.06 ± 0.369^cB^	6.42 ± 0.090^bA^	19.88 ± 0.345^aB^	**	**	**
	14	20.65 ± 0.409^bA^	6.50 ± 0.320^cA^	25.26 ± 0.725^aA^	**	**	**
Intestinal digestion							
CUPRAC	1	2.67 ± 0.401^cB^	25.83 ± 0.575^aB^	22.08 ± 1.769^bB^	**	**	**
	14	53.66 ± 3.126^aA^	41.33 ± 0.822^bA^	37.04 ± 0.629^bA^	**	**	**
DPPH	1	12.61 ± 1.285^aA^	10.26 ± 0.465^bA^	13.68 ± 1.295^aA^	**	NS	**
	14	9.58 ± 0.520^bB^	10.99 ± 1.163^bA^	13.55 ± 0.144^aA^	**	NS	**
FRAP	1	1.34 ± 0.010^bA^	1.74 ± 0.026^aA^	1.37 ± 0.021^bA^	**	**	**
	14	1.35 ± 0.021^bA^	1.55 ± 0.023^aB^	1.32 ± 0.012^bA^	**	**	**
TPC	1	0.00 ± 0.000^cB^	15.38 ± 0.346^aA^	12.72 ± 0.285^bB^	**	**	**
	14	19.68 ± 0.238^aA^	16.05 ± 0.601^bA^	19.90 ± 1.607^aA^	**	**	**

*Note*: Means with different lowercase and uppercase letters are significantly different within a row (among the samples) and within a column (among storage days) (*p < .05; **p ≤ .01; NS, nonsignificant) *S* = Samples; *D* = Storage days; *S* × *D* = Interaction between the samples and storage days.

Abbreviations: KC, plain kefir, KK, kefir‐based smoothie with kale; KS, kefir‐based smoothie with spinach.

It was found that the TAC and TPC analyses in gastric and intestinal digestion stages were statistically significant (Table 5). Regarding sample type, as a result of in vitro gastric digestion analysis, the smoothie with spinach showed higher TAC and TPC values than other samples. Especially, for the control sample (KC), it was found that the TAC and TPC values were determined higher than a predigestion step; on the contrary, those of smoothies with kale were lower. After in vitro gastric digestion, the KS samples showed higher DPPH and TPC content in comparison to the initial values. Similar findings were reported by Ribeiro et al. (2021) who reported a decrease in the TPC values of yogurt incorporated into olive pomace during the intestinal process. The higher TAC values of control kefir during the gastric process conditions might be explained by the following approaches affecting the antioxidant mechanisms: (i) lactoferrin, serum albumin, casein, uric acid, beta‐carotene, and retinol composition of kefir (Cervato et al., 1999; Perna et al., 2014; Stinco et al., 2022); (ii) amino acids (phenylalanine, methionine, cysteine, tryptophan, etc.) and bioactive peptides derived from kefir protein (Aloglu & Oner, 2011; Gamba et al., 2020; Power et al., 2013); (iii) the scavenging/reducing of reactive oxygen species and iron of starter microbiota (Biadała & Adzahan, 2021; Liu et al., 2005; Parrella et al., 2012; Yilmaz‐Ersan et al., 2018); and (iv) lipophilic components such as conjugated linoleic acid and liposoluble vitamins (vitamin E) released in acidic conditions (Badr El‐Din & Omaye, 2007). Furthermore, various mechanisms between milk proteins (casein and whey proteins) and polyphenolic components such as hydrogen bonds, ionic, hydrophobic interactions, and van der Waals forces can result in lower TAC and TPC values of smoothies with vegetables during acidic conditions of gastric digestion (Ozdal et al., 2013; van de Langerijt et al., 2023; Yildirim‐Elikoglu & Erdem, 2018). Especially, hydrogen and/or covalent bindings can be formed between polar groups of casein such as an amino group of lysine and a carboxylic acid group of aspartic and glutamic acids and polar groups of antioxidant compounds (Tosif et al., 2021). It was thought that the interactions between the dissolved kefir components and metabolites formed by the starter culture may have caused the loss of copper‐reducing activity, specifically, low CUPRAC values at the gastric stage. When comparing the storage days, it was found that the gastric digestion CUPRAC, DPPH, and TPC values of control and the smoothie with spinach samples increased, whereas the values of the smoothie with kale were similar (*p* ≤ .01). Generally, the control sample (KC) exhibited stronger DPPH radical scavenging activity and a reduction from ferric iron (Fe^3+^) to ferrous iron (Fe^2+^) during the gastric step. Similarly, in a study by Ustun‐Aytekin et al. (2020), it was also found that the DPPH values of undigested and gastric‐digested kefirs were 4.20% and 63.06%, respectively, whereas the FRAP values decreased during the gastric digestion step. However, Abd El‐Fattah et al. (2017) found that in milk‐digested pepsin (0.01%), the DPPH and FRAP values were 73.42% and 0.193%, respectively. In comparison to the other studies, the differences in TAC values during the digested process might be related to the in vitro‐digested processes (e.g., temperature, incubation time, enzyme, and pH).

After the in vitro intestinal process, significant differences were established among the samples according to the TAC methods and TPC values. In vitro intestinal CUPRAC values were determined in the KK samples on the first day of storage, whereas the control had the highest value at the end of storage. In vitro intestinal DPPH values were established higher for the KS samples, while the KK had the highest FRAP values which might probably be due to the emerging iron‐chelated components in the kale. pH changes in the analysis environment may cause the transformation of some phenolics to pro‐oxidants and degradation or conversion of some phenolics, as the antioxidative compounds released during assay may increase, decrease, or maintain. In this context, electron donation assessed by the FRAP can demonstrate variations (Cuvas‐Limon et al., 2022; Ydjedd et al., 2017). In vitro intestinal TPC values were identified in the KK sample on the first day of storage, whereas the KS and control had the highest values at the end of storage. During the intestinal procedure, the released amino acids, peptides, and phenolic compounds contributed to the higher TPC values of the digested samples. Furthermore, based on the Folin–Ciocalteu method used in this study, not only the free polyphenolics but also nonphenolics such as proteins, amino acids, residual sugars, organic acids, and other hydrophilic components could affect the analysis. Furthermore, the hydrophobic bonds, van der Waals complexes, and hydrogen bonds are formed between the polyphenols and proteins and/or peptides depending on the enzymatic, acidic, oxidative, and thermal conditions of the medium. Polyphenols and protein interactions caused increased TAC and TPC values in the gastric medium with pepsin and low pH, whereas unstable changes occurred in the intestinal medium with pancreatin and neutral pH. Simulated digestion TAC results of the present study revealed that gastric condition produced antioxidant derivatives as a result of hydrolysis of phenolics and proteins as in the KC and KS samples. In contrast, the neutral pH of the intestinal medium caused losses of antioxidant capacity probably because of the unstable intestinal medium and the degradation of antioxidants. Our results are in agreement with those reported by Rodríguez‐Roque et al. (2014) who identified a decrease in the hydrophilic and lipophilic antioxidant activities of blended fruit juice–milk beverages that decreased between 11% and 36% in the small intestinal digesta. El‐Messery et al. (2021) also highlighted that the antioxidant compounds of milk beverages were more reactive particularly in the gastric conditions (acidic pH) than in the intestinal conditions (neutral pH) probably due to the high release of bioactive, with scavenging activities, under the acidic medium. Helal et al. (2022) revealed that the antioxidant capacity (the ABTS and FRAP) of the in vitro‐digested coffee‐fortified yogurt sample was significantly lower than that of digested plain yogurt. Similar findings were confirmed by some other researchers, who suggested that the analysis medium (e.g., enzymes, pH, and oxidation) and complexes formed or antagonistic interactions among the bioactive components or with other food constituents could contribute to the loss in the antioxidant capacity (Durmus et al., 2021; Simonetti et al., 2021; Yildirim‐Elikoglu & Erdem, 2018). However, more studies should be conducted in an attempt to reveal the interaction between the milk components and antioxidative compounds during the gastrointestinal digestion step.

### 3.4 Bioaccessibility indexes of the samples

The bioavailability of functional compounds is the most important factor demonstrating the release, stability, and metabolization of food nutrients by the human intestinal cells. Therefore, in order to estimate the functional effect of foods, bioavailability and bioaccessibility indices are evaluated. Bioavailability analysis provides information about the amount of nutrients passing into the systemic circulation and involves human and animal trials. In contrast to bioavailability, bioaccessibility helps to establish the amount of the functional bioactives released in the model system including the oral, gastric, and intestinal conditions. In the present study, in vitro bioaccessibility analyses of the CUPRAC, DPPH, FRAP, and TPC were carried out by simulating the digestive system conditions on the first and final days of storage (Table 6). The bioaccessibility index was calculated using the values identified initially and after the gastric and intestinal digestion. It was revealed that there emerged significant differences (*p* < .05) among the bioaccessibility indexes of the samples (Table 6). KC had the highest bioaccessibility indexes in both gastric and intestinal steps, whereas KK had the lowest bioaccessibility indexes. In line with our findings, Simonetti et al. (2021) reported that the bioaccessibility indices were well above 100% for goat yogurt samples fortified with *Rhus coriaria* leaf powder except for gallic acid, rutin, and epicatechin. In contrast to our study, Oliveira and Pintado (2015) reported that the bioaccessibility indices of peach‐ and strawberry‐enriched yogurt were 20% and 53%, respectively. Stinco et al. (2022) revealed that the content of milk fat in milk–mandarine beverages increased the bioaccessibility in vitro of phenolic compounds (*p* < .05), while a moderate fat level (1.7%) caused the highest bioaccessibility for bioactive carotenoids. Rodríguez‐Roque et al. (2014) stated that the addition of milk to fruit juice resulted in a decreased bioaccessibility of phenolics, hydrophilic compounds, and vitamin C, whereas there was an increase in the bioaccessibility of lipophilic compounds (carotenes and xanthophylls). It was calculated that the bioaccessibility values of milk fermented by five *B. longum* subsp. *longum* strains were between 175% and 358% (Gagnon et al., 2015). Durmus et al. (2021) reported that the bioaccessibility of the antioxidants was less than 25% in yogurts with black mulberry acidified by both bacterial culture and glucono‐d‐lactone. As mentioned above, the highest bioaccessibility indices of control may be associated with several factors related to (i) the release of bioactive peptides with antioxidative properties from kefir, (ii) the effect of starter microorganisms, (iii) the formed structural changes such as degradation and deglycosylation in nutritional components, (iv) reversible and/or irreversible complexes between polyphenols of vegetables and kefir components (protein, lipids, amino acids, vitamins, and minerals), (v) enzyme activity, temperature, and pH changes in model system, and (vi) structural and biological changes in polyphenols as a result of chemical, oxidative, polymeric, and enzymatic reactions occurring during simulated gastrointestinal digestion. Some researchers pointed out that bioaccessibility indices of plant‐fortified dairy products demonstrated an increase and/or decrease based on the individual phenolics such as flavonols, catechins, and gallic acids, lipophilic and/or hydrophilic attributes, fiber content, and the protocols used in assays (Helal et al., 2022; Kostić et al., 2021; Milinčić et al., 2022; Papierska et al., 2022; Tosif et al., 2021).

**TABLE 6:** Bioaccessibility indexes of the samples.

Bioaccessibility index	Days of storage	Samples			Significance		
		KC	KK	KS	*S*	*D*	*S* × *D*
Gastric							
CUPRAC	1	113.04 ± 4.609^aB^	31.14 ± 3.486^cA^	76.21 ± 1.399^bB^	**	**	**
	14	158.34 ± 3.065^aA^	40.17 ± 3.352^cA^	128.25 ± 2.531^bA^	**	**	**
DPPH	1	316.73 ± 35.199^aB^	100.60 ± 7.869^bA^	130.53 ± 15.348^bA^	**	**	**
	14	543.31 ± 38.359^aA^	93.39 ± 2.667^bA^	136.33 ± 5.059^bA^	**	**	**
FRAP	1	439.15 ± 59.701^aB^	13.06 ± 1.035^bB^	69.27 ± 6.460^bA^	**	**	**
	14	1728.27 ± 257.611^aA^	42.33 ± 6.721^bA^	83.38 ± 13.110^bA^	**	**	**
TPC	1	59.88 ± 8.886^bB^	31.98 ± 1.967^cA^	124.42 ± 2.853^aB^	**	**	**
	14	254.48 ± 9.827^aA^	37.35 ± 2.841^cA^	205.00 ± 7.178^bA^	**	**	**
Intestinal							
CUPRAC	1	10.87 ± 1.571^bB^	50.52 ± 0.936^aB^	59.93 ± 4.002^aB^	**	**	**
	14	247.05 ± 17.858^aA^	97.09 ± 0.175^cA^	150.01 ± 2.556^bA^	**	**	**
DPPH	1	502.16 ± 44.679^aA^	119.54 ± 4.848^bA^	202.88 ± 15.197^bA^	**	**	NS
	14	392.85 ± 51.805^aA^	109.21 ± 12.454^bA^	139.83 ± 1.189^bB^	**	**	NS
FRAP	1	236.57 ± 26.714^aB^	14.65 ± 0.086^bB^	28.91 ± 2.762^bA^	**	**	**
	14	710.37 ± 46.432^aA^	19.47 ± 1.757^bA^	40.97 ± 3.749^bA^	**	**	**
TPC	1	0.00 ± 0.000^bB^	76.52 ± 1.855^aB^	79.62 ± 2.002^aB^	**	**	**
	14	242.42 ± 5.413^aA^	92.19 ± 4.993^cA^	161.70 ± 17.077^bA^	**	**	**

*Note*: Means with different lowercase and uppercase letters are significantly different within a row (among the samples) and within a column (among storage days) (*p < .05; **p ≤ .01; NS, nonsignificant) *S* = Samples; *D* = Storage days; *S* × *D* = Interaction between the samples and storage days.

Abbreviations: KC, plain kefir; KK, kefir‐based smoothie with kale; KS, kefir‐based smoothie with spinach.

### 3.5 Principal component analysis (PCA)

**FIGURE 2:**
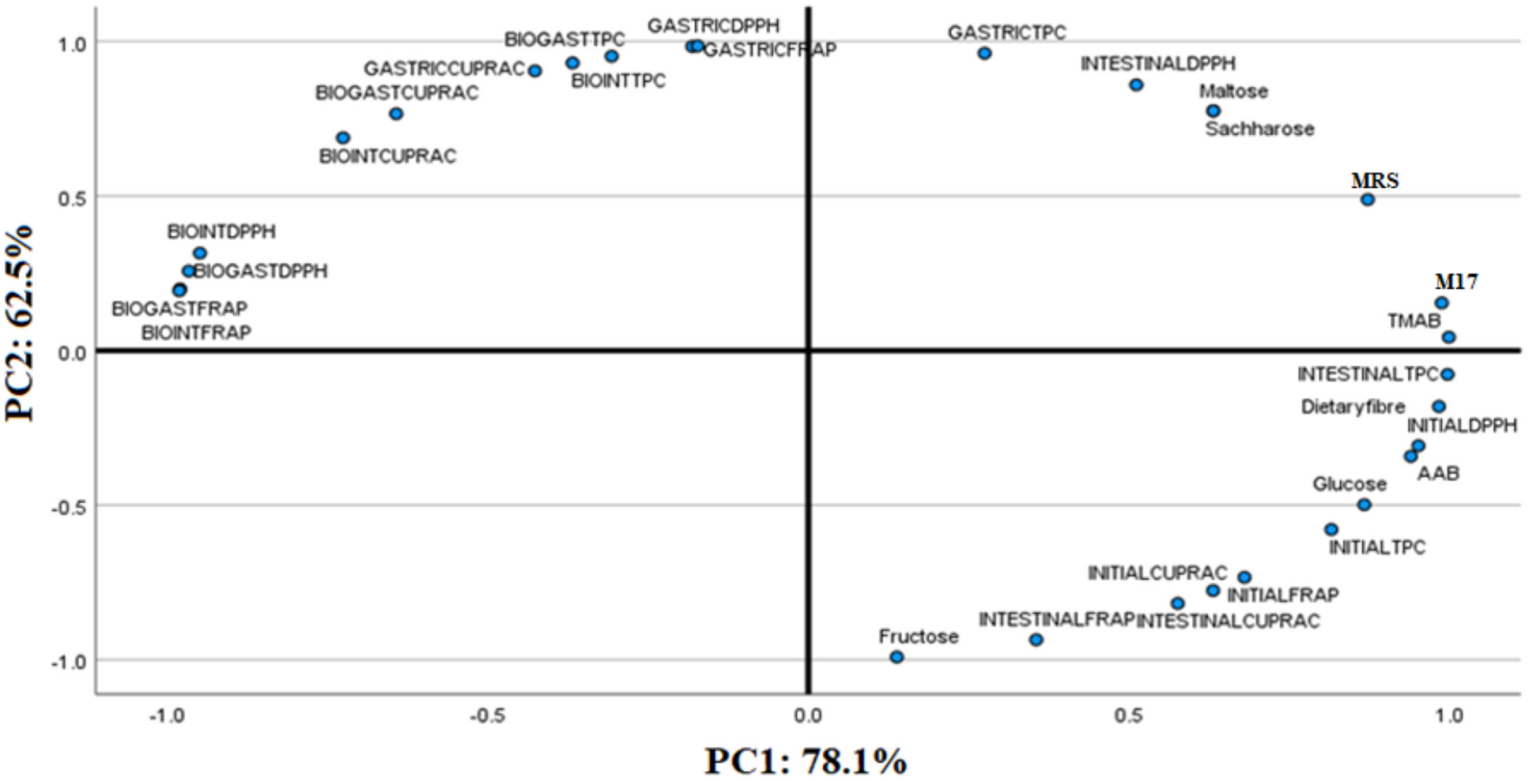
Principal component analysis of the relationships among the analyzed attributes (INITIAL; before digestion procedure, GASTRIC; after the gastric digestion procedure; INTESTINAL; after the intestinal digestion procedure; BIO, bioaccessibility index).

PCA is used to describe similarity/diversity among the attributes analyzed and to visualize the possible interrelation among them. In this study, PCA was conducted to cluster the relationships among analyzed attributes (Figure 2). While PC1 explained 78.1% of the total variance, PC2 accounted for 62.5%. PCA score plot demonstrated a clear separation into three groups according to their similarities. The TMAB counts, Lactobacillaceae counts on MRS agar, Lactobacillaceae counts on M17 agar, maltose and saccharose contents, and TPC values obtained after the gastric digestion step and DPPH values after the intestinal digestion step were located on both PC1‐ and PC2‐positive axis. DPPH, FRAP, and CUPRAC values obtained after the gastric digestion step and bioaccessibility indexes were separated on both the negative side of PC1 and the positive side of PC2. It might mean that bioaccessibility indexes could affect more in vitro gastric assay. PC1 on the positive axis was highly influenced by the contents of dietary fiber, glucose, and fructose, the counts of acetic acid bacteria, and TAC and TPC values identified from initial and after intestinal digestion procedures. As expected, dietary fiber, an antioxidative compound, was located closer to initial TAC values of samples. PCA has confirmed results obtained from the present study showing that smoothies formulated with/without different vegetables have different parameters.

## 4 CONCLUSION

In recent years, the smoothies prepared with different foods have been increasing in both industrial and home production. The present study aimed to evaluate some quality, antioxidative, and in vitro digestion properties of kefir‐based smoothies with fortified kale and spinach. Results obtained revealed that all properties of smoothies were significantly influenced by the kefir and vegetable formulation. There were some significant differences within the counts of microbial growth throughout the storage. Fortification of kefir with kale and spinach enhanced some components such as carbohydrates, dietary fiber, and sugar compounds of samples and led to an increase in their initial antioxidative properties. After the gastric digestion step, the sample with kale had the lowest TAC and TPC values. In vitro intestinal FRAP values were higher for the sample with kale, while the sample with spinach had the highest DPPH values. The bioaccessibility indexes of plain kefir were calculated higher in comparison to the samples with kale and spinach. Therefore, further studies are needed to identify the interactions between the fermented milk components and antioxidative compounds in the gastrointestinal tract, and in vivo bioavailability mechanism should be carried out.

## AUTHOR CONTRIBUTIONS

**Lutfiye Yilmaz‐Ersan:** Conceptualization (equal); investigation (equal); methodology (equal); project administration (equal); supervision (equal); writing – original draft (equal); writing – review and editing (equal). **Tulay Ozcan:** Investigation (equal); methodology (equal); validation (equal); writing – review and editing (equal). **Buse Usta‐Gorgun:** Data curation (equal); formal analysis (equal); investigation (equal); software (equal); validation (equal). **Melike Ciniviz:** Data curation (equal); formal analysis (equal); software (equal); validation (equal). **Gokce Keser:** Data curation (equal); formal analysis (equal); investigation (equal); validation (equal). **Ilay Bengu:** Data curation (equal); investigation (equal); software (equal). **Raziye Asli Keser:** Formal analysis (equal); investigation (equal).

## FUNDING INFORMATION

This study was supported by the Bursa Uludag University Uludağ Üniversitesi.

## CONFLICT OF INTEREST STATEMENT

The authors declare that there are no conflicts of interest.

## Supporting information


Data S1:

## Data Availability

Data obtained within the article will be made available on reasonable request.
