# Supplementary material for: Bioaccessibility and antioxidant capacity of kefir‐based smoothies fortified with kale and spinach after in vitro gastrointestinal digestion
Source: Food Sci Nutr. 2024 Jan 8;12(3):2153–65. doi: 10.1002/fsn3.3917 (PMC10916544; doi:10.1002/fsn3.3917)
Supplement: Supplementary file 1 — Data S1: [file FSN3-12-2153-s001.docx]

**R^2^ and chromatograms for fructose, glucose, saccharose and maltose and for samples**

| Parameter | Fructose | Glucose | Saccharose | Maltose |
| --- | --- | --- | --- | --- |
| Rt | 7,7 | 8,1 | 8,9 | 11,2 |
| LOD | 5,5 | 3,9 | 4,8 | 3,9 |

**S1. Fructose**

**S2. Glucose**

**S3. Saccharose**

**S4. Maltose**


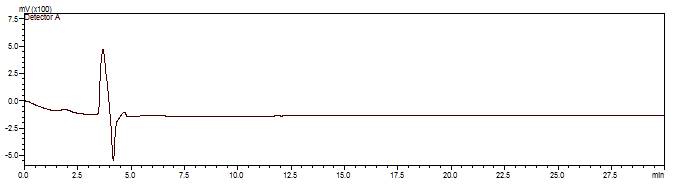


**S5. Chromatogram of sample**

**Total Antioxidant Capacity (TAC) and Total Phenolic Content (TPC) assays**


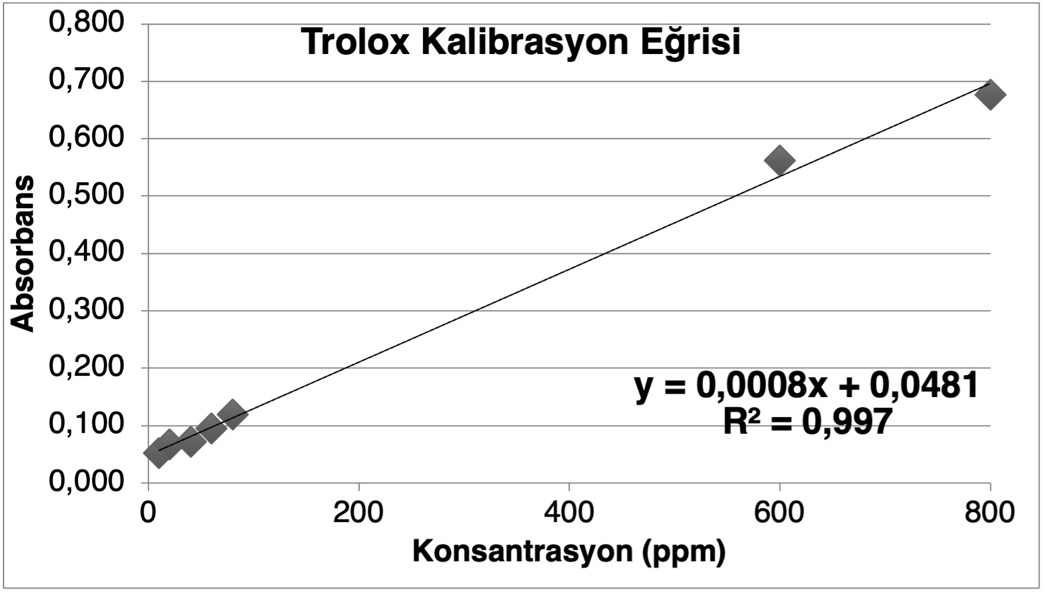


**S6. Calibration curves and equations with R^2^ for CUPRAC assay**


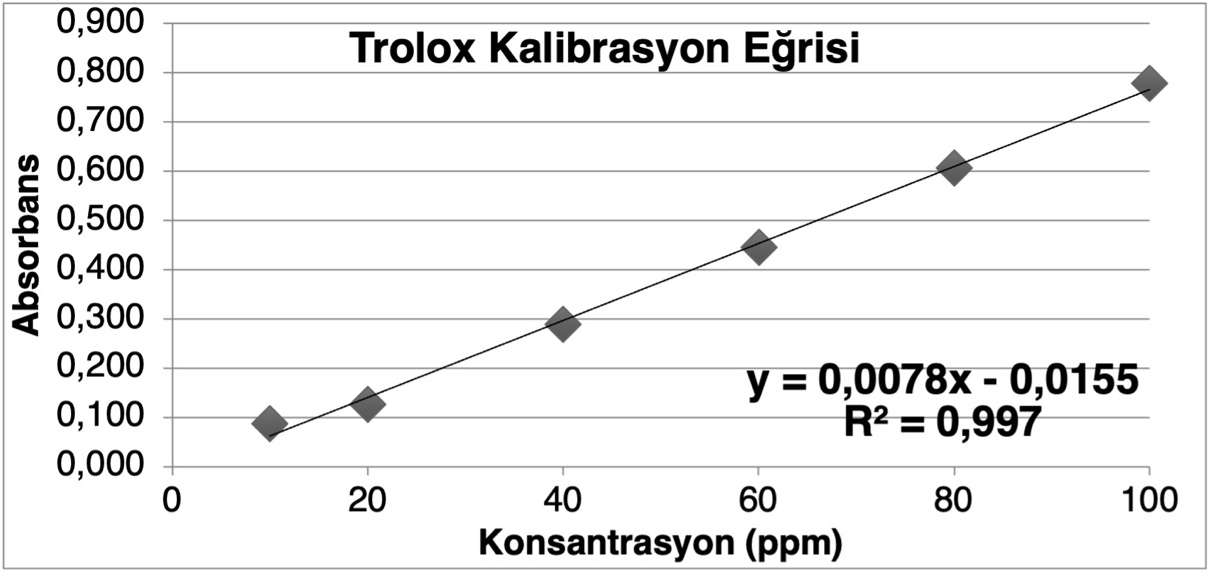


**S7. Calibration curves and equations with R^2^ for DPPH assay**


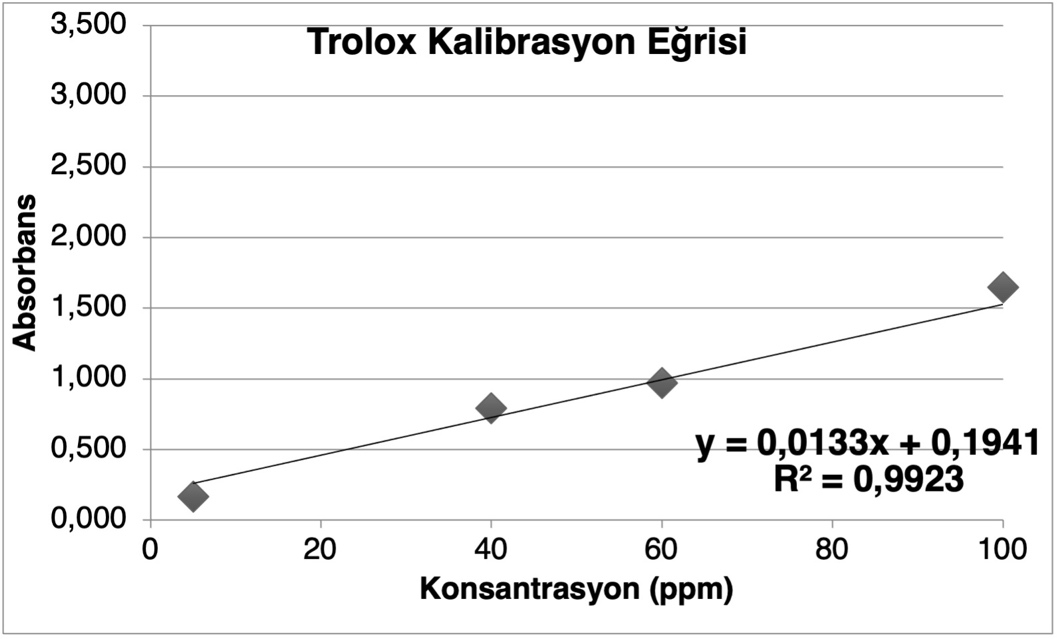


**S8. Calibration curves and equations with R^2^ for FRAP assay**


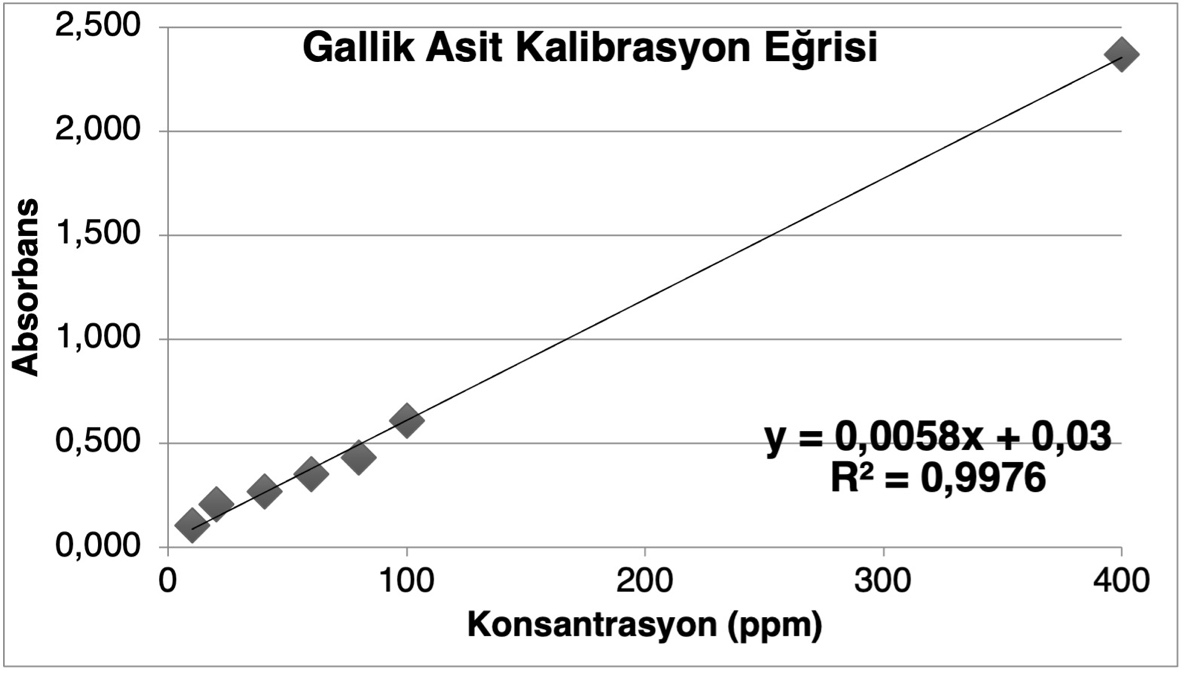


**S9. Calibration curves and equations with R^2^ for Total Phenolic Content (Gallic acid) assay**
